# Using genomics to combat infectious diseases on a global scale

**DOI:** 10.1186/s13059-015-0822-y

**Published:** 2015-11-19

**Authors:** Amy K. Cain, John A. Lees

**Affiliations:** Wellcome Trust Sanger Institute, Wellcome Trust Genome Campus, Hinxton, Cambridge CB10 1SA UK

## Abstract

A report on the seventh annual Infectious Disease Genomics Conference, held in Hinxton, Cambridge, UK, 14–16 October 2015.

## Meeting report

Organized as part of the Wellcome Genome Campus Advanced Courses and Scientific Conferences, the Infectious Disease Genomics conference (IDG) is an intimate conference of 120 infectious disease and genomics experts from around the globe, and this year it was held in the newly renovated Wellcome Genome Campus Conference Centre. The work presented spanned novel applications of genomics to human health, as well as aspects of mathematics, web development, molecular biology, and classic phylogenetics to target a range of pathogens: bacteria, viruses, and parasites. Despite this broad scope, the talks successfully came together to address how genomic techniques can be used to increase understanding of the transmission, spread, and evolution of disease-causing agents.

## Visualization of large and complex datasets

Current genomic datasets of infectious diseases contain orders of magnitude more samples than studies from the first time this meeting took place. Combined with increasingly sophisticated and high-resolution epidemiological data, the need to process and visualize these sorts of data in an intuitive way has become clear and some elegant solutions were presented at IDG 2015.

David Aanenesen (Imperial College London, UK, and Wellcome Trust Sanger Institute, UK) gave an impressive live demonstration of a variety of bioinformatics tools that his group have developed. In one example, assembled sequence data and metadata are uploaded through a web browser, and then are rapidly analyzed, returning an interactive and dynamic interface to allow the user to explore phylogenies in the context of global population structure.

We learned that people travel further and more often than their ancestors, and they almost all have mobile phones. This was the starting point for Caroline Buckee’s (Harvard University, USA) presentation, showing that data from mobile phone providers can be used to track human movement. By combining this with disease surveillance data, maps can be produced to predict and interpret disease transmission at city-level resolution.

Phelim Bradley (University of Oxford, UK) showcased the Mykrobe antibiotic resistance predictor. Although the back-end uses the technique of mapping reads to a variation graph to avoid reference bias, this is completely hidden from the user. Instead, a polished drag-and-drop interface usable on a laptop presents results in a simple format in a matter of minutes. A test set of phenotypically typed *Staphylococcus aureus* and *Mycobacterium tuberculosis* isolates showed that Mykrobe’s predictions of resistance were extremely accurate.

Although phylogenetics is a staple of the field, an effective way of comparing different trees that can be obtained from the same dataset has been lacking. Caroline Coljin (Imperial College London, UK) covered her development of such a metric, which has been distributed as an R-package that allows simple visualization of an incredibly large multidimensional space, and which can highlight key variable points in existing tree topologies.

## Novel approaches to analysis of sequence data

Both larger sample sets and innovative wet-lab methods have driven the development and application of new analysis methods, though in these talks the focus on application to real datasets and interpretation of results was not lost.

Genome-wide association studies (GWASs), a well-established approach in the human genetics field, have only recently been performed on haploid organisms. The conference proved that the infectious disease community is continuing to refine their association studies, with three talks in separate sessions adopting the approach. Philip Kremer (AMC Amsterdam, the Netherlands) showed that clustering populations of 96 *Listeria monocytogenes* genomes on a phylogeny was sufficient to discover associations between disease severity and several sequence variations. Claire Chewapreecha (University of Cambridge, UK) used an alignment-independent approach of testing sequence words instead of single nucleotide polymorphisms to find variants in an accessory gene of *Burkholderia pseudomallei* associated with distinct geographical presentation of disease.

In rapidly mutating viruses such as HIV, assembly of single sample genomes from deep population reads is difficult, but possible. Christophe Fraser (Imperial College London, UK) showcased the method his group has developed for this purpose, which can be used to create an alignment of single genomes that will increase power in a viral-load GWAS. Direct application of GWAS techniques to diverse HIV samples was also shown to be possible by Robert Power (Africa Centre for Population Health, South Africa).

By sequencing samples to a high depth, subtle genomic changes can be detected, such as variations in prevalence of co-infecting subpopulations. The genetics of diverse viral populations was explored in the vector stage of the dengue virus by Shuzhen Sim (Genome Institute of Singapore, Singapore). She investigated the population bottleneck that occurs in transmission from infected human to mosquito from a transmission and evolutionary perspective. Innovative research by Josephine Bryant (University College London, UK) used phasing of short reads and longitudinal deep sequencing to determine the number of co-circulating strains in a cytomegalovirus superinfection and discover how they compete with each other.

## Drug resistance and classic phylogenetics

As with any infectious disease conference, there was mention of drug resistance in many talks, as well as a dedicated session to antimicrobial resistance. The first talk of the conference from Cesar A. Arias (University of Texas, USA) set the scene, where multiply antibiotic-resistant hospital pathogens in Latin America were recognized as a serious public health threat. Using comparative genomics, he outlined the changes of the population genetics of *S. aureus* in Latin America and the emergence of a new dominant epidemic strain (USA300 Latin American Variant, USA300-LV). The potential role of a unique mobile genetic element likely involved in heavy metal resistance in the emergence and spread of USA300-LV was highlighted. Roy Kishony (Israel Institute of Technology, Israel) used deep sequencing of *Burkholderia dolosa* isolates within patients over time to show that multiple adaptive mutations in the same genes can rise in frequency, but never fully take over the population. Furthermore, multiple parallel lineages were shown to exist within patients over several years. This study illustrates the need to routinely sequence infectious disease samples deeply and look for minority variants within the data.

Talks on antibiotic resistance using molecular epidemiology included Teemu Kallonen (Wellcome Trust Sanger Institute, UK) tracking the emergence of antibiotic-resistant *Escherichia coli* strains across the UK over a 10-year period, to examining methicillin-resistant *S. aureus* microevolution and transmission within a single hospital over a year, as described by Francesc Coll (University of Cambridge, UK).

## Genomics of non-typical pathogens

Three sessions were dedicated to parasitology, vectors, and viral infections. Mostly these were key pathogens in emerging infectious diseases within the developing world.

The Keynote lecture delivered by Jean-Michel Claverie (CNRS-Aix-Marseille University, France) was described several times throughout the conference as “mind-blowing”. He introduced the fascinating and confusing world of giant viruses, a field that has developed over the past 10 years. We learned that these viruses can reach up to 1.5 μm in length (larger than some parasites), contain a 2.8 Mb genome, and have 2556 coding sequences, only <10 % of which have homology to any other sequences in current databases.

Transcriptomics opens a window to understanding complex parasite infections. Zbynek Bozdech (University of Singapore, Singapore) took an interesting approach to investigating *Plasmodium falciparum* pathogenesis by defining a transcriptomic profile characteristic of asymptomatic infections. Compared to symptomatic infections, genes associated with the G0 and G1 stage of eukaryotic cell differentiation were upregulated, suggesting that a novel stage of development occurs in these parasites: a resting G0/G1 state. Sally Warring (New York University, USA) used transcriptomics to characterize expression patterns of transposable elements, which make up an unusually high proportion of the *Trichomonas vaginalis* genome.

The Pf3k Consortium aims to sequence and call variation of 3000 *P. falciparum* genomes and provide these data as a community resource. Gil McVean (University of Oxford, UK) presented five separate areas that different groups of the consortium are working on. PacBio sequencing was highlighted as being instrumental to obtain high-quality reference genomes in diverse regions of the genome. Other work included more sensitive variant calling with graph-based mapping, and removing the effect of recombination from population structure-based analysis. Two talks focused on studying vectors of malarial parasites, with Daniel Neafsey (Broad Institute, USA) and Martin Donnelly (Liverpool School of Tropical Medicine, UK) using comparative genomics to understand the capacity and insecticide resitance of malarial vectors.

The final talk of the conference from Annette MacLeod (University of Glasgow, UK) focused on estimating the genetic diversity of *Treponema gambiense*, which causes the severe and underdiagnosed disease sleeping sickness across the tsetse belt of Africa. MacLeod’s phylogeny of 78 diverse gambiense genomes revealed that subtype 1 of gambiense was separate to subtype 2, representing an asexual, mitotic reproducing successful clonal lineage and potentially new species.

## Concluding remarks

Jane Carlton (New York University, USA) summed up the conference noting the high diversity of talk topics and commended the outstanding work of the presenters. Within IDG 2015, there was an overall feeling of excitement and optimism that we are developing a range of novel tools that we are starting to successfully apply to combat infectious disease globally.

## Abbreviations

GWAS: Genome-wide association study
IDG: Infectious disease genomics conference
Pacbio: Pacific bioscience

